# Improving event prediction using general practitioner clinical judgement in a digital risk stratification model: a pilot study

**DOI:** 10.1186/s12911-024-02797-5

**Published:** 2024-12-18

**Authors:** Emma Parry, Kamran Ahmed, Elizabeth Guest, Vijay Klaire, Abdool Koodaruth, Prasadika Labutale, Dawn Matthews, Jonathan Lampitt, Alan Nevill, Gillian Pickavance, Mona Sidhu, Kate Warren, Baldev M. Singh

**Affiliations:** 1 School of Medicine, Keele University, University Road, Keele, Staffordshire, ST5 5BG UK; 2 New Cross Hospital, The Royal Wolverhampton NHS Trust, Wolverhampton, WV10 0Q UK; 3 Faculty of Education, Health and Wellbeing, University of Wolverhampton, Gorway Rd, Walsall, WS1 3BD UK; 4 The City of Wolverhampton Council, Civic Centre, St. Peters Square, Wolverhampton, WV1 1SH UK; 5 School of Medicine and Clinical Practice, Faculty of Science and Engineering, University of Wolverhampton, Wolverhampton, WV1 1LY UK

**Keywords:** Mortality, Urgent care, Risk prediction, Global clinical judgement, General practitioner

## Abstract

**Background:**

Numerous tools based on electronic health record (EHR) data that predict risk of unscheduled care and mortality exist. These are often criticised due to lack of external validation, potential for low predictive ability and the use of thresholds that can lead to large numbers being escalated for assessment that would not have an adverse outcome leading to unsuccessful active case management. Evidence supports the importance of clinical judgement in risk prediction particularly when ruling out disease. The aim of this pilot study was to explore performance analysis of a digitally driven risk stratification model combined with GP clinical judgement to identify patients with escalating urgent care and mortality events.

**Methods:**

Clinically risk stratified cohort study of 6 GP practices in a deprived, multi-ethnic UK city. Initial digital driven risk stratification into Escalated and Non-escalated groups used 7 risk factors. The Escalated group underwent stratification using GP global clinical judgement (GCJ) into Concern and No concern groupings.

**Results:**

3968 out of 31,392 patients were data stratified into the Escalated group and further categorised into No concern (*n* = 3450 (10.9%)) or Concern (*n* = 518 (1.7%)) by GPs. The 30-day combined event rate (unscheduled care or death) per 1,000 was 19.0 in the whole population, 67.8 in the Escalated group and 168.0 in the Concern group (*p* < 0.001). The de-escalation effect of GP assessment into No Concern versus Concern was strongly negatively predictive (OR 0.25 (95%CI 0.19–0.33; *p* < 0.001)).

The whole population ROC for the global approach (Non-escalated, GP No Concern, GP Concern) was 0.614 (0.592—0.637), *p* < 0.001, and the increase in the ROC area under the curve for 30-day events was all focused here (+ 0.4% (0.3–0.6%, *p* < 0.001), translating into a specific ROC c-statistic for GP GCJ of 0.603 ((0.565—0.642), *p* < 0.001).

**Conclusions:**

The digital only component of the model performed well but adding GP clinical judgement significantly improved risk prediction, particularly by adding negative predictive value.

**Supplementary Information:**

The online version contains supplementary material available at 10.1186/s12911-024-02797-5.

## Background

An estimated 20% of unplanned or emergency admissions [1], may be preventable by primary care intervention [2]. A systematic review of risk identification tools for emergency admissions identified 27 data-based risk engines [3] while a cross-sectional survey found 39 tools deployed across UK primary care [4]. Examples include, the Patients At Risk of Rehospitalisation (PARR) tool [5, 6] and Combined Model in England [7], the Scottish Patients at Risk of Readmission and Admission (SPARRA) in Scotland [8] and Predictive Stratification Model (PRISM) in Wales [9]. Generally, they focus on population stratification for non-elective care, draw on similar data sets and have recognised limitations [7, 10].

Using data driven risk prediction models for identifying those at risk of unplanned admission and who may have complex care needs has shown some success, particularly with the addition of information from the GP clinical system [3]. Despite this, reliance on thresholds of risk may escalate large numbers for assessment that will not have an adverse outcome [11] and the intent to improve care can increase emergency admissions, length of stay and use of services [9, 12, 13]. There is limited evidence from systematic reviews, on whether active case management of these high-risk groups leads to a reduction in unscheduled urgent care use [13, 14] and some studies have found an increase in admissions following certain interventions, for example, hospital at home [14]. Analysing studies included in these reviews further reveals that methods used to identify people for case management were not robust, ranging from hierarchical condition category models based on health insurance data [15] and those in a hospital setting receiving home assistance [16].

Global clinical judgment (GCJ) for risk identification is often disparaged and is rarely captured systematically [11, 17], even though clinical judgment is essential in the diagnostic pathway [18]. Emergent evidence suggests that clinical judgment can be used effectively for risk prediction particularly when ruling out disease [19, 20] and predictive accuracy improves when data driven risk and clinical judgment are combined [21, 22].

Evidence suggests that implementing a system where people are identified as at risk of unplanned admission utilising both data driven methods and GP GCJ, and escalating for active case management those who would benefit the most from existing community resources using multi-disciplinary approaches would improve quality of care, reduce unplanned admissions, reduce burden on health services using available resources, reduce healthcare costs by up to 15%, improve patient satisfaction and treatment adherence [23]. In a previous study we described the potential resource requirements for those with escalated care needs [24]. Evidence suggests that patients who are EOL or whose acute conditions are receptive to intensive primary care management, for example cellulitis, dehydration, Chronic Obstructive Pulmonary Disease and pneumonia have better outcomes when identified through risk prediction models [3].

The development of a patient level live integrated data system, “Wolverhampton GP Triage”, for a whole practice population, has led to a system that integrates risk factor driven stratification with GCJ to refine the risk assessment of patients who may access urgent care services or be at risk of mortality. This incorporates an approach to prior and post probability analysis of binary decision making at the risk factor driven step and then, sequentially, of clinical judgement, a methodology in line with the seminal paper of Gill et al*.* “Why clinicians are natural Bayesians” [25].

The purpose of this pilot study was to explore the performance of a risk stratification system that combined selected clinical parameters recorded in the electronic health record and clinician judgement, to predict non-elective care and mortality.

## Methods

### Study design and setting

Prospective whole population cohort study set in a UK city amongst 6 participating GP practices (without selection criteria or financial or other incentive). GP practices were invited to take part via their Primary Care Network (PCN). Six volunteered. There were no other selection criteria and no financial or other incentive. One GP from each practice was involved in pilot study design and undertook the clinical assessment of risk factor escalated patients. All 6 GPs were senior, established partitioners qualified for > 10 years.

### Data

The established Wolverhampton Integrated Clinical Data Set links primary care, hospital, and community services data under GDPR regulation [26]. Demographic variables included age, sex, ethnicity, and the Index of Multiple Deprivation (IMD) ranked score. Ethnicity data from all sources were reviewed, only unambiguous data were accepted, then recoded into White, South-Asian, Black, Mixed Ethnicity, Chinese or Unknown. The morbidities utilised were the 16 most common long-term conditions in the population (Supplement 1). The variables chosen for digital risk stratification were based on common variables used in other risk prediction tools and assessed in preliminary work to be linked to emergency activity and mortality. The seven risk factors were: ≥ 3 Accident and Emergency (A&E) attendances to Royal Wolverhampton NHS Trust (RWT) over the prior 12 months (defined as emergency department attendances not leading to a hospital admission); ≥ 3 non-elective admissions (NEA) over the previous 12 months to RWT; the 30-day emergency admissions predictor PARR score at a threshold value of 80% [5, 6]; ≥ 3 comorbidities, the electronic frailty index (EFI) moderate or severe classification, nursing home residency and end of life registration (EOL).

### Outcomes

Accident and Emergency (A&E) attendance, non-elective admissions and mortality (also summated as “Any event”) at 30 and 90 days from study start point (time of GP clinical assessment). Mortality was determined from hospital mortality statistics and rolling NHS Strategic Tracing Service checks.

### GP Clinical assessment

Patients underwent initial first tier digital risk stratification into Escalated and Non-escalated groups. Allocation into the Escalated group required at least one positive risk variable. Those in the Escalated group underwent further second tier stratification by GP clinical assessment using their GCJ into Concern and No Concern groupings. All data and risk stratification was presented in a live interactive clinical system as a “dashboard” with links to the patients’ electronic records as required. Allocation into No Concern or Concern groupings was intentionally not defined nor imposed proscriptively, but rather the intent was to capture GCJ using Bayesian methodology [25, 27]. In formative clinical dialogue, the emergent concept of Concern related to whether patients had unmet clinical need, were clinically unstable, might require non-elective emergency care, were in the last year of life, or would benefit from a multi-disciplinary team (MDT) process. The prospective objective was to consider the associations and performance metrics of GP GCJ. Of the 3,968 escalated, 492 (12.3%) patients did not undergo rapid assessment by their study GP within the stipulated time frame and were thus reallocated to the No Concern category.

### Statistical method

All data were analysed on IBM SPSS version 26. When comparing independent groups, Student’s t-test and the Chi-square test were used for the difference between means and proportions, respectively and Analysis of Variance (ANOVA) was used to compare multiple group means. Analysis of independent factors with the binary dichotomised risk escalation outcomes of 30-day and 90-day No Event versus Any event was by binary logistic regression with Odds Ratios (OR) and their 95% confidence intervals. Receiver-operating analysis determined the performance metrics of the model. Principle components analysis (at an Eigen value of ≥ 1 and with rotation) was deployed to consider the inter-dependence of independent variables. The results are presented as the mean ± SD or as percentages. The statistical significance of all tests applied was set at* p* < 0.05.

### Patient and public involvement

None.

## Results

### Population characteristics of data driven, and clinical judgement risk stratified groupings.

**Fig. 1:**
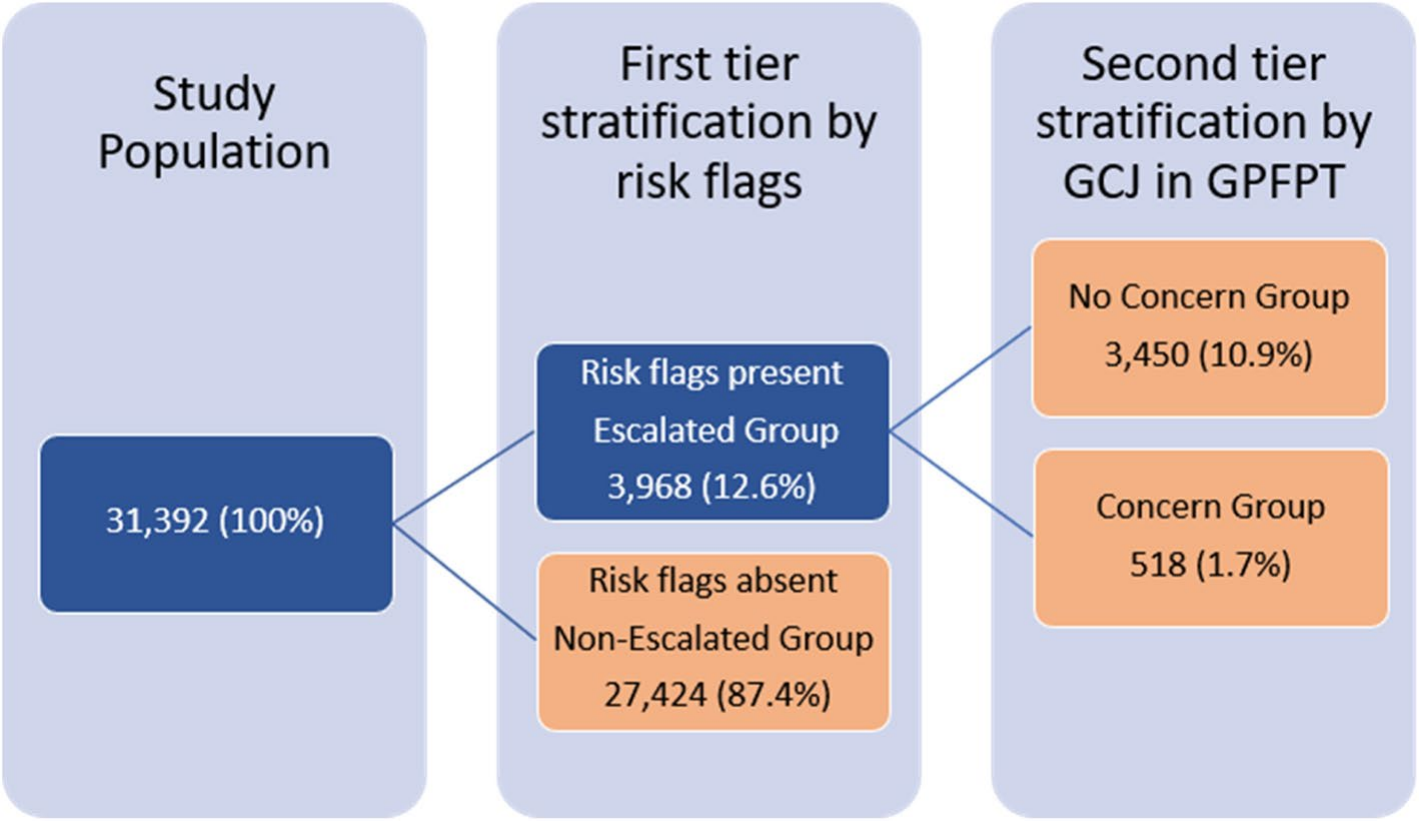
The dispersion of the study population into risk stratified groupings, first by data driven risk flags and second by Global Clinical Judgment (GCJ) in GP First Point Triage (GPFPT) resulted in 3 final cohorts (see Table 1)

The 6 practices had a population of 31,392 (mean 4633; SD 1011; range 3956 to 6973). A schema for the study’s risk stratification protocol is shown in Fig. 1 with clinical characteristics of various groupings given in Table 1. Each tier of stratification was older (F = 4554.3, *p* < 0.001), had higher preceding A&E attendances (*X*^*2*^ = 2,041.9, *p* < 0.001), higher preceding non-electives admissions (*X*^*2*^ = 1,284.6, *p* < 0.001) and was increasingly clinically complex (*X*^*2*^ = 23,825.1, *p* < 0.001).

**Table 1:** Characteristics of those not identified by risk factors and therefore not escalated, and of those escalated then triaged by GP clinical judgment into No Concern v Concern. Statistical analysis of the 3 groups was by ANOVA or Chi square tests for the comparison of means or proportions, respectively

	**Not escalated**	**Escalated, GP No Concern**	**Escalated, GP Concern**	**3 Group comparison**
	*n* = 27,424 (87.4)	*n* = 3, 450 (10.9)	*n* = 518 (1.7)	
Age (years): mean (SD)	36.0 ± 21.2	68.1 ± 17.7	78.3 ± 13.9	F = 4554.3, *p* < 0.001
Gender (male)	51.6	44.9	44.4 *	*X*^*2* ^= 63.1, *p* < 0.001
Ethnicity (white)	53.5	70.9	69.7 *	*X*^*2*^ = 283.1, *p* < 0.001
IMD: mean (SD)	30.5 ± 15.7	26.7 ± 14.7	26.9 ± 14.8 *	F = 104.8, *p* < 0.001
A&E attendances > = 3 in 12 months	0	7.1	8.7 *	*X*^*2*^ = 2,041.9, *p* < 0.001
Non-elective admission > = 3 in 12 months	0	2.9	8.30	*X*^*2*^ = 1,284.6, *p* < 0.001
PARR score > = 80%	0	2.8	11.00	*X*^*2* ^= 31,237, *p* < 0.001
Comorbidities > = 3	0	77.2	84.60	*X*^*2*^ = 23,825.1, *p* < 0.001
Electronic Frailty Index (moderate or severe)	0	43.1	64.50	*X*^*2*^ = 13,737.1, *p* < 0.001
On End-of-Life register	0	7.8	26.40	*X*^*2*^ = 4,069.9, *p* < 0.001
Nursing home resident	0	12.5	15.3 *	*X*^*2*^ = 3,597.8, *p* < 0.001
Any risk flag	0	all	all	-
Total number of flags (SD)	-	1.5 ± 0.8	2.2 ± 1.1	F = 297.1, *p* < 0.001

Figures are column percentages unless otherwise stated. IMD Indices of Multiple Deprivation; A&E Accident and Emergency; PARR Patient’s At Risk of Re-admission

Post hoc analysis for between group differences were all *p* < 0.001 except where * indicated non significance for GP No Concern vs Concern

### The relationship of each tier in the model to prospective events

At both 30- and 90-days, each increasing tier of stratification had higher adverse event rates for A&E attendances, NEA and death (all *p* < 0.001). These outcomes, summated as “Any event” was statistically significant (*p* < 0.001) for each tiered group in the binary logistic regression analysis (Table 2). Odds Ratios (95% CI) for the model, either unadjusted or adjusted are for demographic variables (Tables 1 and 2). In both unadjusted and adjusted analysis the two groups were significantly delineated at both 30- and 90-days.

**Table 2:** Outcomes of the integrated 2 step, 3-tiered risk stratification model for predicting the 30-day and 90-day urgent care and mortality events, summated as “Any event”. Analysis is by binary logistic regression. Odds ratios (95% CI) show either unadjusted or adjusted for demographic variables * (Table 1). Post hoc 2 group comparison for GP Concern vs No concern is also given

			**Odds Ratios (95% CI) (comparator = 1.0)**	
	**Count (%)**	**30-day Any event (%)**	**Unadjusted model,** **X2 = 322.5, *p* < 0.001**	**Adjusted model,** **X2 = 328.7, *p* < 0.001**
**Not escalated**	27,424 (87.4)	520 (1.9)	1.0	1.0
**Escalated, GP No Concern**	3, 450 (10.9)	182 (5.3)	2.9 (2.4—3.4), *p* < 0.001	3.3 (2.7—4.1), *p* < 0.001
**Escalated, GP Concern**	518 (1.7)	87 (16.8)	10.4 (8.2—13.4), *p* < 0.001	12.1 (9.0 -16.4), *p* < 0.001
**GP Concern vs No Concern**	-	-	3.6 (2.8—4.8), *p* < 0.001	4.1 (3.0—5.4), *p* < 0.001
		**90-day Any event (%)**	**Unadjusted model,** **X2 = 478.5, *p* < 0.001**	**Adjusted model,** **X2 = 328.7, *p* < 0.001**
**Not escalated**		1,472 (5.4)	1.0	1.0
**Escalated, GP No Concern**		446 (12.9)	2.6 (2.3—2.9), *p* < 0.001	3.1 (2.7—3.6), *p* < 0.001
**Escalated GP, Concern**		146 (28.2)	6.9 (5.7—8.4), *p* < 0.001	8.4 (6.7—10.6), *p* < 0.001
**GP Concern vs No Concern**			2.6 (2.1—3.3), *p* < 0.001	3.0 (2.4—3.7), *p* < 0.001

^*^Demographic variables (ORs) included in the adjusted model: at 30 days age 0.994 (0.990 – 0.998),*p* < 0.01, gender ns, ethnicity ns, deprivation score 1.005 (1.000 – 1.010), *p* < 0.05; at 90 days age 0.993 (0.991 – 0.996), *p* < 0.001, gender ns, ethnicity ns, deprivation score 1.005 (1.002 – 1.008), *p* < 0.00

Despite low event rates, in each case, compared to the first stage 2 group risk factor partitioning (Non-Escalated vs Escalated), GP GCJ at the 3-group second stage (Non-Escalated vs GP No Concern vs GP Concern) improved specificity, positive predictive value and accuracy whilst retaining strong negative predictive value (Table 3).

**Table 3:** Model performance metrics at the first and second stages of stratification for both the 30- and 90-day summative “Any event” outcome

Model performance metric	1st stage, 2 groups, Non-Escalated vs Escalated	2nd stage, 3 groups, Non-Escalated, GP No Concern, GP Concern	2nd stage, 2 groups, GP Concern vs No Concern
**30-day Any event**			
ROC c-statistic	0.610 (0.598—0.632), *p* < 0.001	0.614 (0.592—0.637), *p* < 0.001	0.603 (0.565—0.642), *p* < 0.001
Sensitivity	34.1%	11.0%	32.3%
Specificity	87.9%	98.6%	88.4%
PPV	6.7%	16.7%	16.8%
NPV	98.1%	97.7%	95.7%
Accuracy	86.6%	96.4%	84.5%
**90-day Any event**			
ROC c-statistic	0.586 (0.572—0.600), p < 0.001	0.588 (0.574—0.602), p < 0.001	0.568 (0.542—0.595), p < 0.001
Sensitivity	28.6%	7.1%	24.7%
Specificity	88.5%	98.7%	89.0%
PPV	15.0%	28.3%	28.1%
NPV	94.6%	93.7%	87.1%
Accuracy	84.5%	92.7%	79.4%

### General practitioner GCJ in the higher risk cohort and prospective events

To examine in more detail the effect of GP GCJ, a separate analysis was undertaken in the Escalated group (*n* = 3,968), subdivided by Concern (*n* = 518) vs No concern (*n* = 3,450) groups. The ORs for the specific comparison of GP Concern vs No Concern emphasisse the strong impact of GP GCJ (*p* < 0.001) (Table 2).

**Fig. 2:**
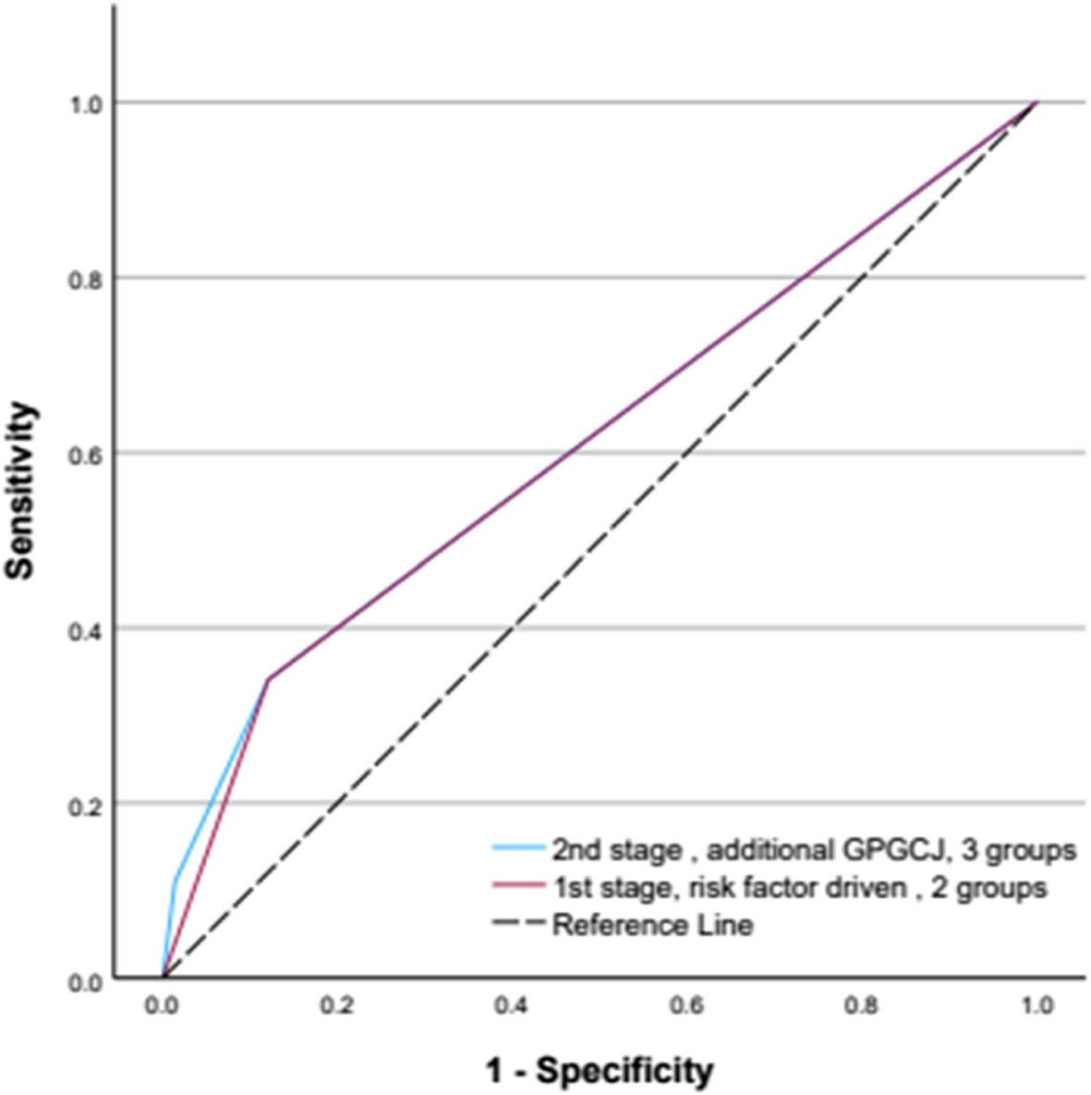
The ROC curves of the 2 stages of the model comparing risk factor stratification only (2 groups) to further stratification by GPGCJ (3 groups) for the prediction of the 30-day summative “Any event” outcome

The ROC performance metrics retained an accuracy of 84.5% and 79.4% for 30- and 90-day events, respectively (Table 3). The whole population global + 0.4% (0.3–0.6%, *p* < 0.001) increase in the ROC area under the curve for 30-day events (Table 3) was all focused here (Fig. 2), translating into a specific ROC c-statistic of 0.603 ((0.565—0.642), *p* < 0.001).

The overall event rate in the whole risk escalated group was 67.8 / 1000, subdividing to 52.7 / 1000 and 168.0 / 1000 with GP GCJ No Concern and Concern, respectively. For mortality alone (which is included in the overall event rate), the rate in the escalated group was 12.1/1000, subdividing to 6.7/1000 and 48.3/1000 with GP GCJ No concern and Concern, respectively at 30-days. The relative risk confirmed by the Concern classification was thus significantly higher (2.5 (2.0 – 3.1), *p* < 0.001) as further conveyed by the ORs (Table 2).

However, for the No concern classification the relative risk was significantly lower (0.8 (0.7 – 0.9), *p* < 0.01), yielding an OR of 0.28 ((0.21 – 0.36), *p* < 0.001)). For the Concern group, GP clinical assessment escalated 6 patients for further consideration of care needs for every 1 who had a 30-day event whilst those de-escalated into the No Concern group 1 event occurred for every 19 patients.

### The clinical associations of GP clinical judgement

The nature of the contribution of the GP assessment was explored by principal component analysis (Supplement 2) among the Escalated group (*n* = 3968). Four components emerged with an Eigen value of > = 1, individually explaining 14.7 – 20.7 and an accumulated 68.4% of the common data variance. Within the four components GP GCJ, was grouped with the EFI measure of frailty.

## Discussion

### Summary

The purpose of clinical risk stratification tools is to stratify populations and identify patients using quantitative measures for clinicians to consider impactful interventions to modify avoidable adverse outcomes, where clinicians add further assessment through clinical judgement.

We believe this to be the first risk prediction model that simultaneously considers clinical complexity, urgent care, and EOL prediction and integrates data driven and GP clinical judgment-based assessments. The study highlighted the importance of GP clinical assessment in a fragmented complex care pathway by enumerating the predictive effect of their clinical judgement. The inclusion of GP clinical assessment improved the prediction of 30-and 90-day events compared to the data driven model alone. The key outcomes were the marked improvement in the predictive ability of 30- and 90-day events when GP clinical assessment was added. In addition to contributing positive risk prediction, it should be noted that GP GCJ contributed very strongly to negative prediction (Table 2).

### Strengths and limitations

Limitations include the small scale of the study, and the GP assessors may not be representative. As this was a pilot study, we did not gather information on outcomes related to quality of care, clinical acceptability of the processes nor did we undertake formal validity testing, however we aim to address these in a future study. As this study took place in a real-world setting, 492 patients that were Escalated were not reviewed by their GP by the study end date. Post-hoc analysis revealed this missing data did not significantly impact the results, however it may highlight constraints GPs may face with this risk stratification model. As health outcomes were not studied, we cannot make conclusions on how this model impacts care. Strengths include curation and deployment of integrated data from primary, community, and secondary care sources into a systematic approach to population wide risk stratification and event analysis was prospective. In comparison to other widely used health care risk stratification tools recently evaluated in a systematic review, the predictive ability of this model would be classified as “good” due to the area under the curve and c-statistic being above 0.70 [28].

### Comparison with existing literature

Digital risk stratification tools tend to rely on quantitative data and are used to predict those at risk of various events (for example, unplanned admissions) without incorporating clinical assessment [29]. Tools based on EHR data have been criticised due to their tendency to perform better at population rather than individual levels which is partly due to the completeness of data, availability of data (for example, disease severity is rarely captured), omission of important predictors and modelling decisions, highlighting the importance of clinical judgement when interpreting results of risk calculators [30, 31]. However, it is inconclusive whether data driven tools perform better than clinical judgement alone [32]. Using clinical judgement alone has been criticised for reducing cost-effectiveness by leading to the potential for increased prescribing [33] or increased investigation [11]. Global clinical judgement is therefore disparaged as an unquantifiable “gut feeling”, feeding an inherent belief in the superiority of quantitative measures over erstwhile intuition [21, 34]. In the converse however, if clinical judgement is disengaged from data predication, the self-estimation of a clinician’s own GCJ can lead to reduced trust in data modelled predicated outcomes [11, 20, 24, 33–36]. However, findings from a systematic review highlighted that capture of clinical judgement alongside a risk prediction tool, can improve performance metrics and predictive accuracy [32], as was replicated in our study. Thus capturing GCJ within a probability-based methodology, can positively impact risk prediction, mirroring findings regarding GP clinical judgement in dementia [22].

In this study we elected not to prescriptively define or enumerate the GCJ of Concern vs No Concern. Instead, we involved clinicians in iterative discussion about binary judgement, emphasising the freedom to be wrong. After completion of the project, the study team outlined a scenario-based descriptor, for use in future studies defining GCJ in GP clinical assessment for Concern as: “A patient who is clinically unstable, or has significant unmet health/care needs, or is likely to have an emergency admission in the near future (3 months) or is likely to be in the last year of life”. If we are to move away from GCJ being reduced to intuitive gut feeling, then it is apposite to question the validity of GP clinical assessment. The descriptor we propose yields both face and content validity which is important for clinical acceptance [13, 18, 35]. Whilst the purpose of this phase of the project was not to explore all aspects of the validity of GCJ, some degree of criterion validity arises out of principle component analysis in which GP clinical assessment was independent of several risk escalators but associated with the electronic frailty index and it is tempting to consider the concept of frailty as the core additional component in the GP global view. The concurrent validity of the GP GCJ is verified in the highly statistically significant prediction of the hard end point clinical outcome measures.

### Implications for research and/or practice

Risk stratification models generally predict when something might happen. In our data the greater impact was the negative predictive value of GCJ. This is consistent with the experience of practicing clinicians who might find it hard to say whether someone will be admitted or will die within a defined time frame but may be more confident predicting that such things are unlikely. Our study emphasises the need to move away from a value proposition of GP GCJ predicate on “what will happen” to a numerically more valuable concept of “what will not”, noting studies showing the clinical value of “ruling out” rather than “ruling in” [20].

In our next phase, deploying at a larger scale we wish to consider whether these findings are replicable; how performance metrics shift in a live and iterative digital platform; how wider care needs can be captured in a systematic manner; analysing arising actions and whether they confer measurable benefit. In all risk stratification models, while event rates may be lower in low-risk groups, numerical gearing means that most targeted events are not captured in higher risk cohorts. The Pareto principle, which states that 20% of causes are responsible for 80% of outcomes, might be addressed by refining risk prediction and improving clinical assessment but perhaps most effectively by shifting performance metrics into a live real time and iterative digital platform [36]. The factors associated with measurable variation in GP GCJ risk prediction and whether it is modifiable must be considered. Colleagues in primary care will be concerned about workload implications and, understandably, the potential for inappropriate scrutiny of measurable GCJ and these must be addressed in an evidenced based manner.

Our view is that health care clinicians, service delivery teams and system designers should not exclude, underestimate, or subjugate clinician decision making from risk prediction without proper consideration.

## Conclusion

This pilot study highlighted that precision of a risk stratification tool to predict escalating urgent care needs and mortality, using EHR data can be improved with the addition of GP global clinical judgement in an NHS UK primary care setting. Further testing of this model is required to ensure findings are replicable at a larger scale.

## Supplementary Information

Supplementary Material 1.

Supplementary Material 2.

## Data Availability

No datasets were generated or analysed during the current study.

## Abbreviations

A&E: Accident and Emergency
EFI: Electronic frailty index
EHR: Electronic Health Record
EOL: End of life
GCJ: Global Clinical Judgement
GP: General practitioner
IMD: Index of Multiple Deprivation
NEA: Non-elective admissions
OR: Odds ratio
PARR: Patients At Risk of Re-hospitalisation
PRISM: Predictive Stratification Model
ROC: Receiver Operating Curve
SPARRA: Scottish Patients at Risk of Readmission and Admission
