# Supplementary material for: Improving event prediction using general practitioner clinical judgement in a digital risk stratification model: a pilot study
Source: BMC Med Inform Decis Mak. 2024 Dec 18;24:382. doi: 10.1186/s12911-024-02797-5 (PMC11654091; doi:10.1186/s12911-024-02797-5)
Supplement: Supplementary file 1 — Supplementary Material 1. [file 12911_2024_2797_MOESM1_ESM.docx]

**Supplement 1** Variables included in digital risk stratification tool

| **Variable** | **Description** |
| --- | --- |
| Age | - |
| Sex | - |
| Ethnicity | White  South-Asian  Black  Chinese  Mixed ethnicity  Unknown |
| Indices of Multiple Deprivation | - |
| Long term condition | Serious mental health condition e.g. psychosis, psychotic depression, manic depression  Musculoskeletal pain condition (osteoarthritis, rheumatoid arthritis)  Macro vascular disease (CVA, CVD, PVD)  Haemoglobinopathy  Asthma  Congestive Heart Failure  Cancer  COPD  Depression  Diabetes  Hypertension  Atrial Fibrillation  Chronic Kidney Disease  Dementia  Epilepsy  Learning disability |
| Patients At Risk of Re-admission (PARR) | Age  Number of emergency admissions in last year  Hospital discharge in previous 30 days  Whether current admission is an emergency  Index of Multiple Deprivation  History in past 2 years of 11 major health conditions from Charlson Comorbidity Index |
| Electronic Frailty Index (EFI) | Moderate or Severe.  Based on 36 deficits comprising 2000 clinical codes within the primary care electronic health record[1] . |
| Accident and Emergency attendance | ≥3 over previous 12 months  Emergency department attendances not leading to a hospital admission |
| Non-elective admissions | ≥3 over previous 12 months |
| Nursing home resident | - |
| End-of-life register | On an EOL register |
| *1 Clegg A, Bates C, Young J, et al. Development and validation of an electronic frailty index using routine primary care electronic health record data. Age Ageing. 2016;45:353–60.* | |
