# Supplementary material for: Improving event prediction using general practitioner clinical judgement in a digital risk stratification model: a pilot study
Source: BMC Med Inform Decis Mak. 2024 Dec 18;24:382. doi: 10.1186/s12911-024-02797-5 (PMC11654091; doi:10.1186/s12911-024-02797-5)
Supplement: Supplementary file 2 — Supplementary Material 2. [file 12911_2024_2797_MOESM2_ESM.docx]

**Supplement 2:**

Principal component analysis of the prior established digital risk flags among those who were risk factor Escalated for global clinical judgement in the GP clinical assessment (n = 3,968).

The key variables in each component are highlighted.

| **Factor grouping component.** | **1** | **2** | **3** | **4** |
| --- | --- | --- | --- | --- |
| A/E attendances ≥ 3 in 12 months | 0.38 | -0.1 | -0.7 | -0.2 |
| Non-elective admission ≥3 in 12 months | 0.82 | 0.01 | -0.1 | -0 |
| PARR score ≥80% | 0.86 | 0.05 | -0 | 0.08 |
| Comorbidities ≥3 | 0.13 | -0.1 | 0.88 | -0.1 |
| Electronic Frailty Index moderate or severe | -0.1 | -0.2 | -0.1 | 0.84 |
| Nursing home resident | -0.1 | 0.77 | -0.1 | -0.1 |
| On End-of-Life register | 0.1 | 0.79 | 0.07 | 0.12 |
| GP clinical assessment outcome | 0.25 | 0.28 | 0.11 | 0.64 |
